# Congenital Infiltrating Lipomatosis of the Face: Multimodality Assessment through CT-Scan and Magnetic Resonance Imaging

**DOI:** 10.3390/diagnostics14020176

**Published:** 2024-01-12

**Authors:** Giuseppe Cicero, Francesco Marcello Arico, Silvio Mazziotti

**Affiliations:** Department of Biomedical Sciences and Morphological and Functional Imaging, University Hospital Messina, 98124 Messina, Italy; france.arico@gmail.com (F.M.A.); smazziotti@unime.it (S.M.)

**Keywords:** congenital infiltrating lipomatosis of the face, computed tomography, magnetic resonance imaging

## Abstract

Congenital infiltrating lipomatosis of the face is a rare and benign condition involving a hamartomatous proliferation of mature adipose cells. The final diagnosis derives from a combination of clinical data, histology, and imaging features and affects the treatment decision. This report describes the clinical case of a young patient suffering from facial lipomatosis with particular emphasis on radiological findings detected at CT-scan and magnetic resonance imaging.

Congenital infiltrating lipomatosis of the face (CILF) is a rare and benign pathology of unclear origin and characterized by a hamartomatous proliferation of mature adipose cells.

This condition generally occurs as a unilateral, infiltrating and ill-defined adipose lesion characterized by indolent and progressive growth [1,2,3,4].

CILF presents with the hypertrophy of soft and hard structures, leading to facial asymmetry, and is usually associated with dental growth alterations, bone hypertrophy, macroglossia, and parotid gland proliferation [5].

A precise differentiation from other clinical conditions is often challenging and a definitive diagnosis relies on a comprehensive approach, integrating physical, radiological, and histopathological findings [6].

In particular, radiological imaging plays a pivotal role in assessing infiltrative lipomatosis.

While the role of ultrasound (US) can be limited due to the restricted field of view, computed tomography (CT) and magnetic resonance imaging (MRI) are capable of providing crucial information, such as the precise localization and extent of fatty infiltration and coexistent muscle and skeletal involvement [5].

A 20-year-old male patient complaining of significant swelling of the left cheek with facial asymmetry was admitted to our hospital. At clinical examination, marked enlargement involving the middle and the lower third of the left hemiface was noticed without any skin abnormality. On palpation, the mass was soft, painless, and not fluctuating.

This condition was already present during childhood with a progressive worsening that led to surgical intervention with a diagnosis of facial lipoma when the patient was 10 years old.

During the following years the symptoms steadily returned.

After admission to our hospital, contrast-enhanced computed tomography (CT) and magnetic resonance imaging (MRI) were performed for a comprehensive assessment of the underlying soft and bone tissues and potential pre-operatory planning.

MRI showed a diffuse lesion with a fatty signal with no detectable peripheral capsule located in the subcutaneous tissue of the left hemiface with lipomatous infiltration of the ipsilateral parotid gland, while the CT scan allowed a more accurate assessment of the associated bone dysmorphism and teeth abnormalities.

The radiological suspicion of CILF was then confirmed by histology, due to the absence of lipoblasts or malignant features.

CILF is characterized by lipomatous infiltration and bone hypertrophy leading to unilateral hemifacial enlargement [7].

Slavin et al. in 1983 first documented [8] this condition, which can involve different anatomical districts [1].

CILF primarily emerges in the first three decades of life, often presenting during infancy as swelling of cheek or chin. However, the disorder’s phenotypic variability and unclear pathogenesis pose challenges in diagnosis and treatment planning [7].

US, CT, and MRI can collectively contribute in the comprehensive assessment of CILF [5].

US can be hampered by its resemblance to surrounding buccal fat and may find challenges in delineating the extent and infiltration. However, the color-Doppler technique can effectively evaluate vascularization and, in the perspective of differential diagnosis, rule out vascular malformations.

Although valuable in displaying nonencapsulated and low-attenuation masses, consisting in lipomatous infiltration, CT is particularly helpful in detecting skeletal changes [5].

On the other hand, MRI takes advantage of higher soft-tissue contrast than CT and can accurately outline lesion extent and margins [8].

Therefore, information from CT and MRI is often complementary and the two techniques are concurrently performed.

However, despite technical advancements, a definitive diagnosis of CILF is not achievable relying solely on imaging findings [4].

In fact, differential diagnosis of CILF includes several conditions, such as vascular abnormalities, infections or neoplasms [1,2], and the final diagnosis is therefore made through a combination of clinical data, biopsy with histology, and imaging findings [2,3,4].

The treatment is still debated among clinicians, due to unpredictable prognosis, high risk of recurrence, and variable postoperative outcomes [7].

In fact, if an early surgical intervention could prevent further bony and dental malformations, a delayed approach, performed after complete facial development, could allow a definitive final treatment, decreasing the number of procedures and the related risks with a lower occurrence of relapses [1,4].

**Figure 1:**
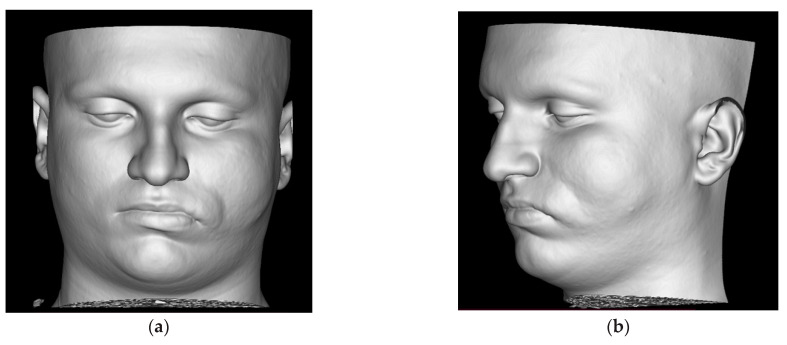

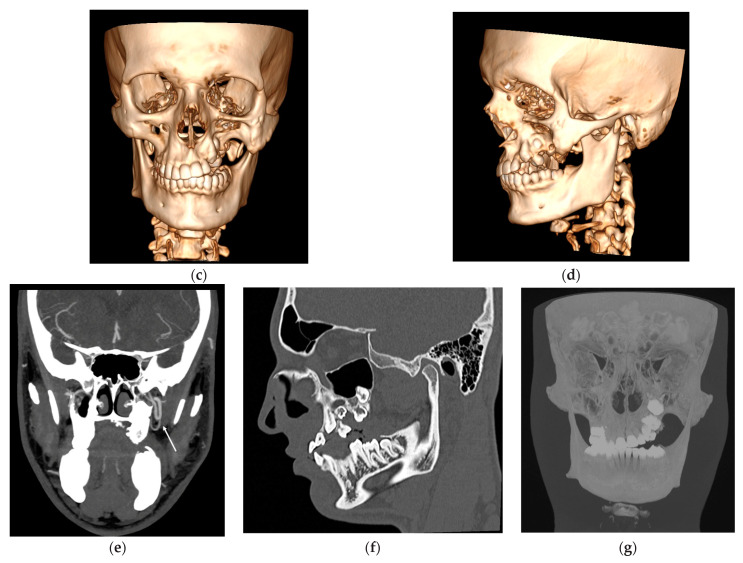
Volume rendering (VR) reconstructions of soft-tissues (**a**,**b**) shows tumefaction of the left hemiface. Bone-based VR images (**c**,**d**) show hyperplasia of the left zygomatic bone and of the ipsilateral hemimandible including the condylar region. Loss of substance in the alveolar process of the left maxillary bone with ectopia of some dental elements, some of which are impacted, are also visible. Coronal maximum intensity projection (MIP) reconstruction (**e**) obtained after intravenous contrast medium injection better demonstrates the ectasia of the left pterygoid venous plexus (arrow). Bone-window sagittal-oblique image (**f**) and coronal MIP thick-slice reconstruction (**g**) demonstrate deformation of the left superior dental arch with some teeth retention but no bone erosion.

**Figure 2:**
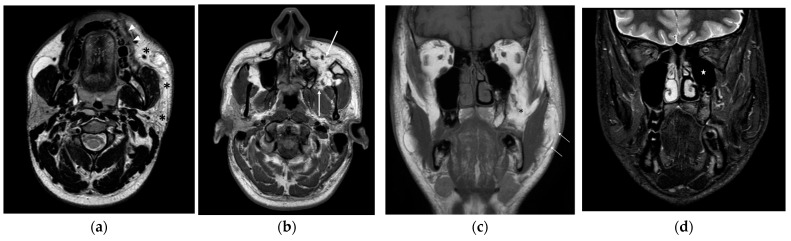
Axial T2-weighted Turbo spin echo scan (**a**) shows a non-capsulated lipomatous lesion, extending into the buccal, masticatory, and parotid spaces of the left hemiface (asterisks) with infiltration of the left orbicularis oris muscle (arrowheads). Axial T1-weighted turbo spin echo image (**b**) also demonstrates the deep extension of the lesion into the infratemporal fossa and pterygomandibular space (spaces between arrows). Coronal T1-weighted turbo spin echo scan (**c**) displays fat hypertrophy within the left hemiface (arrows) and the pterygomandibular space (asterisk). On coronal T2-weighted spectral presaturation with inversion recovery scan (**d**) hypoplasia of the ipsilateral maxillary sinus (star) is also detectable.

## Data Availability

No new data were created or analyzed in this study. Data sharing is not applicable to this article.
